# Folk Knowledge and Perceptions about the Use of Wild Fruits and Vegetables–Cross-Cultural Knowledge in the Pipli Pahar Reserved Forest of Okara, Pakistan

**DOI:** 10.3390/plants13060832

**Published:** 2024-03-14

**Authors:** Sadia Jabeen, Fahim Arshad, Nidaa Harun, Muhammad Waheed, Saud Alamri, Shiekh Marifatul Haq, Ivana Vitasović-Kosić, Kaneez Fatima, Abdul Shakoor Chaudhry, Rainer W. Bussmann

**Affiliations:** 1 Department of Botany, Faculty of Life Sciences, University of Okara, Okara 56300, Pakistanfahim.arshad@uo.edu.pk (F.A.); f19-phd-bot-5013@uo.edu.pk (M.W.); kfokian@gmail.com (K.F.); 2 Department of Botany and Microbiology, College of Science, King Saud University, Riyadh 11451, Saudi Arabia; 3 Department of Ethnobotany, Institute of Botany, Ilia State University, Tbilisi 0162, Georgia; marifat.edu.17@gmail.com (S.M.H.); rainer.bussmann@iliauni.edu.ge (R.W.B.); 4 University of Zagreb Faculty of Agriculture, 10000 Zagreb, Croatia; ivitasovic@agr.hr; 5 Agriculture Building, School of Natural and Environmental Sciences, Newcastle University, Newcastle upon Tyne NE1 7RU, UK; abdul.chaudhry@newcastle.ac.uk; 6 Staatliches Museum für Naturkunde, Erbprinzenstrasse 14, 76133 Karlsruhe, Germany

**Keywords:** wild fruits and vegetables (WFVs), Pakistan, traditional ecology praxis, cross-cultural knowledge, cross-regional differences

## Abstract

Wild fruits and vegetables (WFVs) have been vital to local communities for centuries and make an important contribution to daily life and income. However, traditional knowledge of the use of wild fruits is at risk of being lost due to inadequate documentation. This study aimed to secure this knowledge through intermittent field visits and a semi-structured questionnaire. Using various ethnobotanical data analysis tools and SPSS (IBM 25), this study identified 65 WFV species (52 genera and 29 families). These species, mostly consumed as vegetables (49%) or fruits (43%), were predominantly herbaceous (48%) in wild and semi-wild habitats (67%). 20 WFVs were known to local communities (highest RFC), *Phoenix sylvestris* stood out as the most utilized species (highest UV). Surprisingly, only 23% of the WFVs were sold at markets. The survey identified 21 unique WFVs that are rarely documented for human consumption in Pakistan (e.g., *Ehretia obtusifolia*, *Euploca strigosa*, *Brassica juncea*, *Cleome brachycarpa*, *Gymnosporia royleana*, *Cucumis maderaspatanus*, *Croton bonplandianus*, *Euphorbia prostrata*, *Vachellia nilotica*, *Pongamia pinnata*, *Grewia asiatica*, *Malvastrum coromandelianum*, *Morus serrata*, *Argemone mexicana*, *Bambusa vulgaris*, *Echinochloa colonum*, *Solanum virginianum*, *Physalis angulata*, *Withania somnifera*, *Zygophyllum creticum*, and *Peganum harmala*), as well as 14 novel uses and five novel edible parts. Despite their ecological importance, the use of WFVs has declined because local people are unaware of their cultural and economic value. Preservation of traditional knowledge through education on conservation and utilization could boost economies and livelihoods in this and similar areas worldwide.

## 1. Introduction

Wild fruits and vegetables (WFVs) improve the nutrition, economy, and even cultural identity of people in many regions of the world, especially in rural communities [1]. These plants have one or more parts that, when properly harvested and processed, can be used as food. In developing countries, they are now an integral part of people’s daily lives, serving as food, medicine, raw material for construction, fuel, and shelter [2,3,4]. However, the utilization of these wild resources as part of the human diet is considered one of the most important components of the global food basket [5]. These wild plants can be consumed raw or in the form of herbal teas, sauces, beverages, decoctions, and special dishes [6]. Many wild fruits have different amounts and types of fat, carbohydrates, and proteins compared to cultivated fruits [7]. In addition, they contain important nutrients that are necessary for maintaining health and preventing many diseases [8]. Due to their valuable nutrients, flavor, and aroma, they are also used in times of food shortage [9,10,11].

Household income can also be increased through the production, consumption, and sale of wild fruits, which can be a strong socio-economic factor [5,12]. Therefore, a large number of wild fruits are traded in local, national, and international markets [13]. They are a good source of income for local people who can improve their socio-economic status by promoting culinary tourism. They attract tourists from many parts of the world by promoting an area as a destination for certain types of food [11]. However, significant cultural changes affecting local populations, as well as global climate change, have led to a decrease in reliance on wild plants for food [12]. Pakistan, a developing country, is the eleventh highest-risk country in the world for food insecurity [13]. The use of wild fruits and vegetables as food is an important local survival tactic in times of famine or drought. In order to prioritize the conservation and/or domestication of these plants, it is important to understand the patterns of use of wild fruits and vegetables as well as their cultural significance and economic value [14]. This also has implications for rural development through the commercialization of promising species, as well as for human health through the identification of nutritious species or the promotion of the use of wild fruits.

Many studies have been conducted worldwide on the ethnobotanical and ethnomedicinal aspects of edible wild plants, e.g., in China [15], India [14], Zambia [16], Ethiopia [17], and also in different provinces of Pakistan such as Khyber Pakhtunkhwa (KPK) [6], Balochistan [18], Azad Jammu, Kashmir [19], Gilgit Baltistan [20] and Punjab [21,22,23]. However, no study on the economic utilization of edible wild plants has been conducted in the Pipli Pahar Reserved Forest in the Okara district of the Punjab province. This wild forest was declared a reserve in 2017 to conserve this natural resource. However, some plants (e.g., *Bombax*, *Populus*, *Dalbergia*, *Eucalyptus*, and *Salix*) were cultivated near the forest. Nevertheless, the present study focused only on wild plants as they are able to grow in a natural state (independent of humans, i.e., not cultivated) [24]. It was suspected that there is a variety of WFVs in the area and that local people also use some of these products, but the traditional knowledge of their use was not well-researched or documented. Today, the primary aim of ethnobiological research is to document disappearing traditional knowledge [25]. Against this background, the present study is the first attempt to specifically address the economic use of WFVs by the inhabitants of the Pipli Pahar Reserved Forest. The specific objective of the present study was, therefore, to record and analyze the diversity, distribution, and various methods of WFV use in this forest. In addition, a cross-cultural analysis was conducted with data on similar aspects. The results of this study can serve as a basis for policymakers to identify and implement certain aspects of future research in this area of great importance.

## 2. Results and Discussion

### 2.1. Demographic Details of the Informants

A total of 70 informants, 51.4% men and 48.6% women, took part in the study. The women were reluctant to participate in the interviews as they were usually hesitant to talk to strangers, especially younger people [26]. The women had more knowledge as they were directly involved in the gathering and cooking of these WFVs, in this area. Different ethnic groups participated in the survey; most of them were Qureshi (11.4%), Mughal, and Khokhar (10% each). The majority of the respondents, about 50%, were middle-aged (40–60 years), 36% were young people (20–40 years), and 14% were above 60 years. The older ones had more knowledge than the younger ones, but their less number participated in the survey because of their poor eyesight and memory loos issues. The transfer of knowledge from the old to the young generation seems to be low, as the new generation is less interested in this traditional knowledge. Most respondents were either illiterate (36%) or had only completed elementary school (29%) education. The respondents were a mix of farmers, herders, teachers, forest officers, forest guards, gardeners, landowners, and shopkeepers. However, most information was obtained from shepherds (31%), gardeners (19%), and farmers (17%), as they deal directly with and handle native plants. Farmers and landowners, on the other hand, were mainly aware of the wild species that grow naturally among the crops they cultivate. Some non-resident families of forest guards (7%) and forest officials (3%) who lived in the service buildings also had a good knowledge of forest plants (Table 1).

### 2.2. Diversity of Wild Fruits and Vegetables

The study area has a great floristic diversity. A total of 65 plants were observed to be used as wild food by the inhabitants of the Pipli Pahar area. A total of 65 WFV species belonging to 29 plant families and 52 genera were recorded (Table A1).

A high proportion of these WFVs belonged to the Moraceae family with seven species (11%), followed by Fabaceae and Solanaceae with six species each (9%). The quantitative FIVs (Family Importance Values) also underline the importance of Moraceae (573), Fabaceae (499), and Solanaceae (357) as food plants with the highest values (Table 2). The highest FIVs corresponded to the greater number of informants naming a particular family [27]. Previous studies have also reported that Moraceae is the most important family for wild edible foods in Punjab [21,22]. However, in the Hindu Kush mountain ranges of Pakistan, Moraceae was reported with the least number of edible wild plants [6]. This suggests that the occurrence and utilization of plant species varies from one geographical region to another. However, the number of species in a family is not directly proportional to their use value in a study area. Based on the Family Use Values (FUVs), Arecaceae (5.00), Meliaceae (4.50), and Brassicaceae (4.22) were the most utilized families of WFVs in the study area (Table 2). The plant families with a higher FUV were more frequently used in the community compared to other plant families. It has already been pointed out that different geographical, ecological, and cultural conditions may influence the community configurations of a species at a particular study site. Therefore, the values of FIVs or FUVs for each family may vary by continent, country, or even by region of the same country [28,29,30] (Table 2).

The observations showed that most edible wild plant species were herbaceous (48%), followed by trees (40%), shrubs (11%), and arborescent grasses (1%) (Table A1). Ethnobotanical literature indicates that wild herbaceous have been an important component of human food baskets worldwide [15,31]. The plant species in the Pipli Pahar forest grew indiscriminately in open areas, roadsides, water beds, and cultivated and uncultivated land. It was estimated that most of the species in the study area were wild and semi-wild (66%), and only a few species were cultivated in the cultivated and semi-cultivated environment (34%) (Table A1). In this context, a wild habitat refers to the natural environment, including forests with a comparatively high tree diversity, non-forested ecosystems along water bodies, or open areas where non-domesticated plant species grow spontaneously in self-sustaining populations [15,32,33]. A semi-wild habitat refers to semi-natural ecosystems that are partially tamed or cultivated and whose processes and biodiversity are largely intact, even if they have been altered in intensity or abundance compared to their natural state by human activities, such as fallow land and edges along fields, paths, roads and rivers [15,32,34].

The variety and availability of plants varied with the changing seasons. The local population knew the availability and collection time of WFVs [35]. In general, spring (February–May) and summer (June–October) are the main seasons for collecting WFVs. However, the availability of many plants (especially herbaceous species) also strongly depended on the frequency and amount of rainfall. Plant diversity in the Pipli Pahar area was highest during the rainy seasons, especially during the monsoon season (July to September), as 41 out of 65 species (63%) were available during the monsoon months. Plants such as *Malva parviflora*, which are mainly dependent on rainwater, faded during periods of water scarcity. Therefore, most of the WFVs were not available from December to January (Table A1).

### 2.3. Traditional Utilization Aspects of WFVs

#### 2.3.1. Use Category

**Figure 1:**
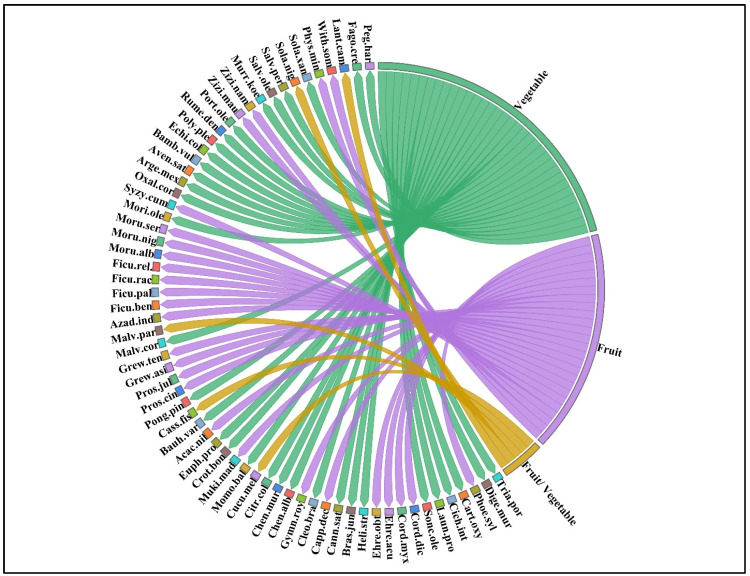
Chord diagram showing the species and type of utilization of wild fruits and vegetables from Pipli Pahar Reserved Forest in Okara, Pakistan. The full names of the species are listed in Table A1 in Appendix A.

In Pipli Pahar, 32 (49%), 28 (43%), and five (8%) species were used as vegetables, fruits, and both fruits and vegetables, respectively (Figure 1). Vegetables were also reported as the most commonly used category in the Jhlem district of Punjab, Pakistan [23]. Due to the different climatic conditions in the Lesser Himalayas, the beliefs and traditions of the locals regarding the use of edible wild plants vary, with the fruits being considered the most edible parts. Another possible reason for these regional differences could be the difference in nutritional value and suitability for use as raw or cooked food.

#### 2.3.2. Most Popular WFVs

The results of this study showed that the RFC was between 1 and 0.01. About 20 plants had the highest RFC values of 1, including *Phoenix sylvestris*, *Cichorium intybus*, *Cordia dichotoma*, *C. myxa*, *Capparis decidua*, *Chenopodium album*, *C. murale*, *Cucumis melo* var. *agrestis*, *Vachellia nilotica*, *Azadirachta indica*, *Morus alba*, *M. nigra*, *M. serrata*, *Syzygium cumini*, *Bambusa vulgaris*, *Ziziphus mauritiana*, *Z. nummularia*, *Solanum nigrum*, and *Physalis angulata*. The plants with the lowest RFC value of 0.04 were *Gymnosporia royleana*, *Croton bonplandianus*, *Euphorbia prostrata*, *Ehretia obtusifolia*, and *Grewia tenax*, followed by 0.01 for *Euploca strigosa* (Table A1 in the Appendix A). The higher RFC values of WFVs showed that these species were popular because the locals were more familiar with these WFVs. The higher RFC values of some species indicate that they are used more by the local population and their knowledge is greater [36]. The UV, on the other hand, indicates the importance of each fruit and vegetable species based on the number of different uses in the community. The highest UV was found in the literature [37] for *Phoenix sylvestris* (5.00), followed by *Azadirachta indica* (4.50) and *Bauhinia variegata* (4.29), indicating that these plants were either frequently used for a specific purpose or for many different purposes [38] by the community. The species with the lowest UV were *Croton bonplandianus*, *Grewia tenax*, and *Lantana camara* (1.00), as they are less utilized by the inhabitants of the Pipli Pahar area.

#### 2.3.3. Edible Parts

**Figure 2:**
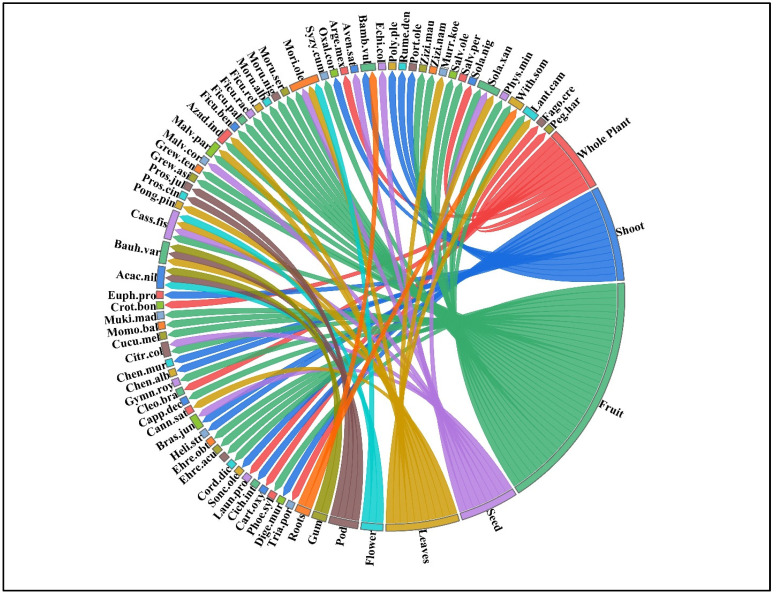
Chord diagram of utilized parts of wild fruits and vegetables from Pipli Pahar Reserved Forest in Okara, Pakistan. The complete names of the species are given in Table 2.

Most indigenous communities were found to use fruits interchangeably with vegetables (39%) as edible parts, followed by sprouts (14%) and whole plants (11%). In contrast, the use of leaves (12%), seeds (9%), pods (5%), roots (4%), and other parts (1% each of soft twigs, buds, and gum) was less common (Figure 2). The results for the use of fruits as the most commonly utilized edible part were found in the older ethnobotanical literature [8,20,39,40,41]. Therefore, it can be said that wild fruits play an important role in meeting the food needs of many rural communities around the world.

#### 2.3.4. Traditional Dishes Recipes of WFVs

**Figure 3:**
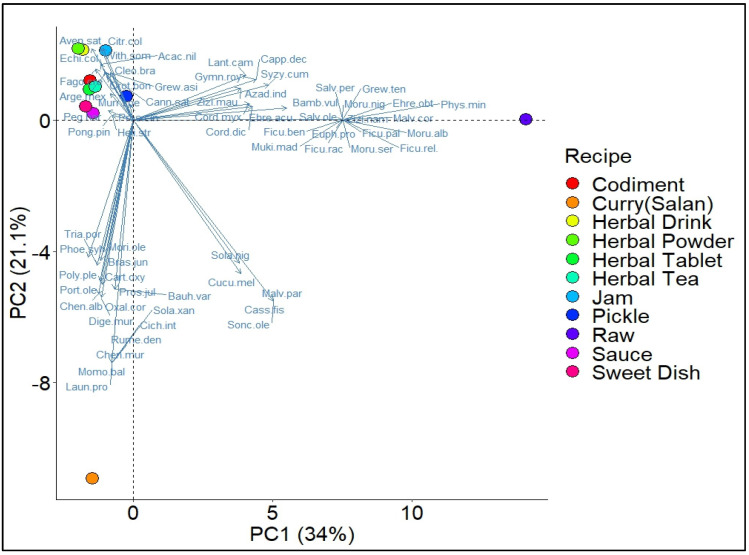
Biplot analysis of Principal Component Analysis (PCA) illustrating the predominant recipes of different plant species in Pipli Pahar Reserved Forest of Okara, Pakistan.

Of the 65 WFVs, 34 (37%) species were eaten raw or uncooked, especially those that had ripened as fruit. Only three (5%) plant species (*Euphorbia prostrata*, *Oxalis corniculata*, and *Bambusa vulgaris*) were found to be consumed raw, while the remaining species were preferably used in cooked form. Most (14%) of the wild vegetable species were cooked into curry (‘salan’). Saag was the most common, well-cooked traditional dish of the region, in which many green wild herbaceous with their sprouts were cooked together with cooking salt and spices. The two main ingredients of the ‘saag’ recipe were *Brassica campestris* and *Spinacia oleracea*, which were boiled in water together with other wild vegetables. The boiled and concentrated product was then fried in oil or butter with onions, garlic, ginger, green chilies, and other spices to make ‘tarka’ (a traditional frying method). Cooking wild vegetable plants together with other cultivated or wild vegetables [42] improved not only the nutritional value but also the flavor of this traditional food of human communities [6]. Besides curry, many (14%) of them were used as pickles, followed by herbal teas (11%) and beverages (9%). In addition, some (6%) WFVs were used to make ‘*chutney*’ (the local name for gravy). Some were also used to make desserts (3%), *murabba* (jam, 4%), herbal tablets, and yogurt (1% each) (Figure 3). Statistically significant differences were found in the use of food plant species for different recipes, represented by axis 1 in Figure 3. A Principal component analysis (PCA) revealed significant differences between the different disorders. PC1 and PC2, characterized by the presence and absence of plant species, respectively, accounted for 55% of the variation in the distribution of species within the biplot.

#### 2.3.5. Nutritional and Medicinal Value of WFVs

**Figure 4:**
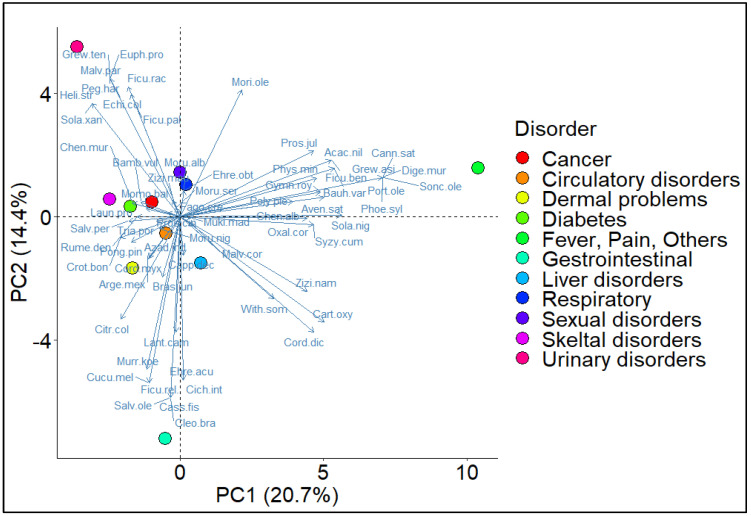
Biplot analysis of Principal Component Analysis (PCA) illustrating the prevalent diseases treated with different plant species in Pipli Pahar Reserved Forest of Okara, Pakistan.

Many WFVs contain large amounts of nutrients, such as fatty acids, minerals, fibers, vitamins, and phenols, which have a high medicinal value [43,44,45,46]. The results showed that most taxa were used to treat fever and pain conditions (21%), followed by gastrointestinal complaints (14%) and other problems (10% each). The lowest number of taxa (2%) was used to treat cancer. As shown in Figure 4, there were statistically significant differences in the use of food plant species for the various ailments defined by axis 1. The principal component analysis (PCA) revealed significant differences between the different complaints. PC1 and PC2, determined by the presence and absence of plant species, respectively, accounted for 35% of the species distribution in the biplot. The analysis revealed 11 disease groups based on the presence or absence of specific species, including circulatory, gastrointestinal, skin, respiratory, venereal, liver, cancer, urinary and skeletal diseases, and fever and pain. The use of edible wild fruits was an essential part of the medical management system for the elderly [2]. Middle-aged or younger people mostly considered the use of phytomedicines as orthodox therapy and preferred to take modern allopathic medicines. Therefore, the traditional knowledge about the medicinal use of wild plants is disappearing more and more [47].

#### 2.3.6. Marketing and Cultivation of WFVs on a Commercial Scale

Integrating WFVs into markets and designing efficient and functional value is key to ensuring their increased availability and utilization [48]. Ultimately, this will have an impact on the food security challenges of today. Despite all the studies that have been conducted over the last 10 years, the full potential of wild edible resources has still not been realized. This is one of the most burning issues that deserves the attention of the scientific community, with special emphasis on the economic benefits and their role in the sustainability of rural areas [49]. In the Pipli Pahar region, almost all WFVs and their products were not considered as saline products. Local people prefer to buy cultivated fruits and vegetables from other farmers or traders but do not even think of marketing their wild food sources. Only 15 species (23%) were sold by the forest department and some locals in the markets of other towns. It was also found that 14 (21%) species of WFV are cultivated in the study area (Appendix A Table A1). A few plants of the *Bauhinia variegata*, *Azadirachta indica*, *Cordia myxa*, *Morus alba*, *M. nigra*, *M. serrata* and *Syzygium cumini*, *Ficus racemosa*, *Moringa oleifera*, *Ziziphus mauritiana*, and *Z. nummularia* species were cultivated by the forest department. *Cassia fistula* and *Phoenix sylvestris* were grown for ornamental purposes in the park, but none of the wild vegetable species were cultivated by the Forest Department of the study area. *Morus serrata* and *Syzygium cumini* were cultivated with other staple cultivated commercial fruit trees, while *Cichorium intybus* was cultivated only as fodder for animals by some native landlords. Poor people who did not own land for farming were not allowed by the forestry department to grow vegetables or fruit on land controlled by the government. However, due to deforestation and urbanization, these wild edible resources could soon disappear. The cultivation and domestication of these WFVs are, therefore, crucial to ensure the conservation of these dwindling plants. This was confirmed by [50], who pointed out that “domestication emerged from food gathering and led almost imperceptibly to cultivation”.

**Figure 5:**
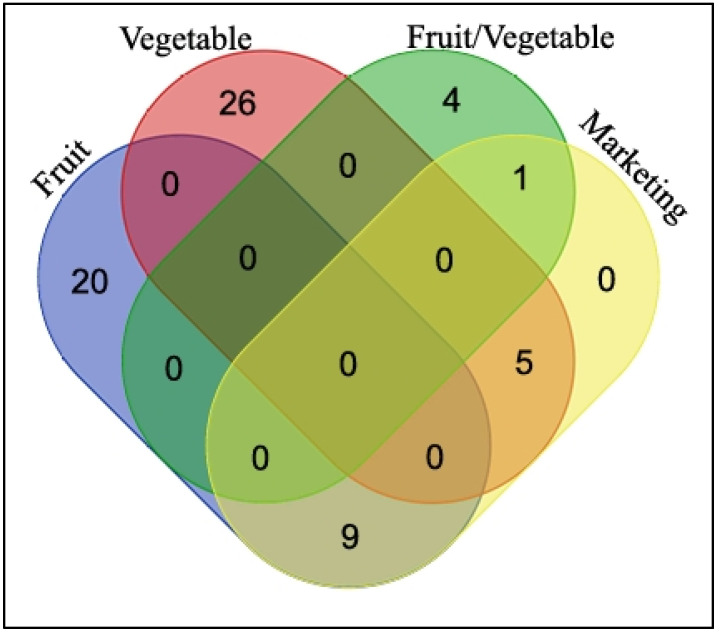
Venn diagram representing unique and common plant species among different categories.

The analysis of the Venn diagram showed that nine fruit plants, five vegetable plants, and only one fruit/vegetable plant were commercialized (Figure 5). The fruits of *Cordia dichotoma*, *C. myxa*, and *Morus serrata* were sold by the forest department in the markets of major cities. The samples of *Chenopodium album* were collected by some women and sold as *saag* in other towns by some local people along with cultivated plants such as *Brassica campestris* and *Spinacia oleracea*. *Capparis decidua* and *Citrullus colocynthis* were also collected by some people for sale in the towns. Other plants such as *Phoenix sylvestris*, *Bauhinia variegata*, *Moringa oleifera*, and *Syzygium cumini* were sold in the local markets, but mainly the park and forest department employees also consumed the products of these species for free. Most of the wild fruit trees were under the control of the forest department, and the people concerned were allowed to consume the fruits without payment. A recent study found that *Bauhinia variegata* and *Chenopodium album* were sold in the markets, as also reported in a previous study, but *Solanum nigrum* was not sold in Pipli Pahar, which was in contrast to a study report on wild fruit data from the Lesser Himalayas region of the Khyber Pakhtunkhwa (KPK) province of Pakistan [42]. This illustrates the influence of regional cultures and traditions on marketing patterns in individual provinces due to ethnic and historical differences.

According to some officials of the Pipli Pahar forest department, the edible wild trees cannot be used for honey extraction, and their wood cannot be used for construction, while these purposes can be fulfilled by non-edible trees. Therefore, the forest department preferred the cultivation of non-edible trees over edible fruit trees. Farmers of the study area also preferred to grow staple crops (such as maize, rice, wheat, potato, mango, banana, orange, guava, etc.) as these species are in demand among consumers and can be sold in the cities at good prices so that they can earn a good profit. However, when they grow WFV crops, they cannot sell them in the markets, as people in the cities do not buy these WFVs because they do not know how to use them. Various reasons, such as lack of government funding, inadequate marketing, lack of value addition, and inability to meet market demand, hindered the cultivation of wild species. Another important factor that influenced the revival and cultivation of WFVs was consumer choice [51].

### 2.4. Impact of Urbanization on the Conservation and Utilization of WFVs

The analysis of the relative frequency revealed alarming facts and findings. Despite their importance, only 25% of the species in the study area were classified as common and abundant, while 20% of the species were classified as rare in terms of abundance. This could be due to urbanization, which has eclipsed the importance of WFVs in changing habits. Only the edible wild species that are also used by city dwellers were classified as important, while lesser-known species were gradually removed. For example, *Momordica balsmina* and *Physalis angulata* (both rare plants) were removed from various locations for road construction, playgrounds, and commercial plant cultivation. A 60-year-old man said that the last time he ate the root of *Launaea procumbens* as a curry (*salan*) was as a child in 1965. After that, the root was never cooked again as it was considered old-fashioned and unpopular due to modernization. If this behavior continues, many WFV species could be neglected or eradicated in the future. Therefore, community education and proper planning are required to conserve and promote the rarely growing edible wild species in the Pipli Pahar region.

### 2.5. Regional Analysis and Novelty Index of Reported WFVs

The national regions of Pakistan are composed of different provinces and ethnic groups living in geographically and climatically diverse areas with different perceptions of different social groups, including dietary habits. The availability of edible wild plants is rooted in the culture and traditions of each area. Factors such as climatic and geographical changes lead to changes in the availability and utilization of edible wild plants and, consequently, their importance as food [52]. Therefore, the documented list of 65 wild fruits and vegetables from the current study was compared with the previous 19 articles on edible wild plants in Pakistan to identify cross-regional differences or overlaps and new aspects of reproduced WFVs. The comparison helps to find out the differences in utilization (edible parts and type of utilization) of indigenous edible wild plants in different regions and communities. These types of differences and similarities are usually measured numerically by ethnobotanists using the Jaccard Index (JI). A higher JI value reflects the similarity of vegetation types in the two areas due to similar geographical or climatic conditions, while lower JI values indicate the opposite [53] (Table 1). In the present study, the JI values ranged from 35.3 to 1.1 (Table 3). The highest value was recorded for the Sargodha district (35.3), followed by Central Punjab (24.3) and the Jhelum district (13.5); all three sites were located in the Punjab province, reflecting the similarity of vegetation. The lowest JI value was calculated in the study by Ishkoman and Yasin in the valleys of Gilgit Baltistan (1.1), where only a single plant similar to the current site was reported. This is due to the fact that there is a major difference between the climate and geography of the two sites, with the reported site being a mountainous region with a cold climate, while the study site is a low land with a warm climate that supports different vegetation growth.

The percentage of similarity or dissimilarity of the edible parts used and the way they are used mainly reflect the differences in the cultural dietary habits of different communities depending on where they live in the different provinces or regions. The current study showed that the highest percentage of similarity of edible parts was used in the Sargodha district (81.5%), followed by D.I. Khan in KPK (72.7%) and Central Punjab (63.0% each). The highest percentage of similarity in the type of use was also found in the Punjab province, i.e., the Sargodha and Central Punjab districts (44.4% each), followed by D.I. Khan in KPK (36.4%) and the Hindu Kush Mountains and Bajur on the Pakistan–Afghanistan border (12.7% each). The Harnai district showed the least similarities in terms of the percentage of edible parts utilized (1.7%) and type of utilization (3.4%). These similarities and differences between the areas are due to geographical, climatic, historical, traditional, and cultural differences between the provinces. The reason for the highest percentage of similarities in the Punjab province is clearly due to the same Punjabi culture in both study areas, i.e., past and present publications. However, the higher percentage of similarities in the other provinces is due to the mixing of culture and traditions of different communities due to many factors such as cross-cultural marriages, tourism, migration for livelihood, and recipe channels on social media. This shows that the national culture of Pakistan is united and cemented in the diversity of socio-cultural thoughts and variations in terms of food habits in the use of WFVs [54,55,56].

**Table 3:** Jaccard index to compare the present study with the published literature of Pakistan to show the novelty aspect of the present study.

Author Citation	Study Area, Province	NP	NPSP	NPDP	NPSM	NPDM	TPCBA	PRAA	PRSA	PPSP	PPDP	PPSM	PPDM	JI	Author Citation
[21]	District Sargodha, Punjab	27	22	2	12	12	24	3	41	81.5	7.4	44.4	44.4	35.3	(Shah et al., 2019)
[22]	Central Punjab	27	17	1	12	6	18	9	47	63.0	3.7	44.4	22.2	24.3	(Shah et al., 2020)
[23]	District Jhelum, Punjab	78	14	3	9	8	17	61	48	17.9	3.8	11.5	10.3	13.5	(Majeed et al., 2021)
[41]	Bajaur Boundary, Pak Afghan Border	63	6	5	8	3	11	52	54	9.5	7.9	12.7	4.8	9.4	(Abdullah & Khan, 2020)
[18]	District Harnai, Baluchistan	59	1	3	2	2	4	55	61	1.7	5.1	3.4	3.4	3.3	(Tareen et al., 2016)
[55]	Ishkoman and Yasin Valleys, Gilgit Baltistan	27	1	0	1	0	1	26	64	3.7	0.0	3.7	0	1.1	(Aziz et al., 2020)
[39]	District Rajouri, Jammu Kashmir	57	6	2	6	2	8	49	57	10.5	3.5	10.5	3.5	7.0	(Rashid et al., 2008)
	D.I. Khan, KPK	11	8	0	4	4	8	3	57	72.7	0.0	36.4	36.4	11.8	Marwat et al., 2011)
[40]	Takhat Sulaiman Hills, KPK	51	3	2	4	1	5	46	60	5.9	3.9	7.8	2.0	4.5	(Ahmad & Pieroni, 2016)
[57]	Division (Malakand, Peshawar, Mardan, D.I. Khan, Bannu, Hazara and Kohat, KPK	25	1	4	3	2	5	20	60	4.0	16.0	12	8	5.9	(Ahmad et al., 2018)
[58]	Kurram District, KPK	55	3	4	4	3	7	48	58	5.5	7.3	7.3	5.5	6.2	(Abbas et al., 2020)
[20]	Chitral, KPK	55	0	2	2	0	2	53	63	0.0	3.6	3.6	0	1.7	(Aziz et al., 2020)
[6]	Hindu Kush Mountain Range KPK	63	5	6	8	3	11	52	54	7.9	9.5	12.7	4.8	9.4	(Abdullah et al., 2021)
[59]	Gadoon Valley	51	4	4	5	3	8	43	57	7.8	7.8	9.8	5.9	7.4	(Khan et al., 2021)
[60]	Different Mountain Packets, KPK	17	3	0	1	2	3	14	62	17.6	0.0	5.9	11.8	3.8	(Shad et al., 2013)
[61]	Lesser Himalayas, KPK, and Punjab	35	6	0	4	2	6	29	59	17.1	0.0	11.4	5.7	6.4	(Abbasi et al., 2013)
[42]	Lesser Himalayas, KPK, and Punjab	45	2	9	2	9	11	34	54	4.4	20.0	4.4	20	11.1	(Abbasi et al., 2013)
[36]	15 different sites in the Lesser Himalayas, KPK, and Kashmir	20	3	2	2	3	5	15	60	15.0	10.0	10	15	6.3	(Abbasi et al., 2013)
[19]	Western Himalaya, Azad Jammu & Kashmir	102	5	7	8	4	12	90	53	4.9	6.86	7.8	3.9	7.7	(Iqbal et al., 2022)

Legends: NP Number of total Plants, NPSP: Number of Plants with Similar Parts used, NPDP: Number of Plants with Dissimilar Parts used, NPSM: Number of Plants with Similar Mode of utilization, NPDM: Number of Plants with Dissimilar or multiple Mode of utilization, TPCBA: Total number of Plants Common in Both Areas, PRAA: Plants Reported in Aligned Areas, PRSA: Plants Reported in Study Area, PPSP: Percentage of Plants with Similar Parts Used, PPDP: Percentage of Plants with Dissimilar Part Used, PPSM: Percentage of Plants with Similar Mode of utilization, PPDM: Percentage of Plants with Dissimilar or multiple Mode of utilization, and JI Jaccard Index.

**Figure 6:**
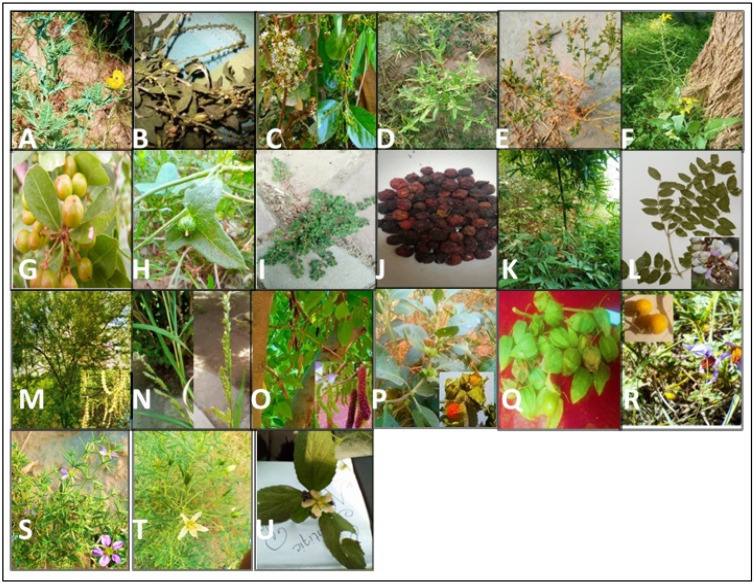
A pictorial representation of some typical WFVs in the studied area of Pipli Pahar where (**A**) *Argemone mexicana* L., (**B**) *Croton bonplandianus* Baill., (**C**) *Ehretia obtusifolia* Hochst. ex ADC., (**D**) *Euploca strigosa* (Willd.) Diane & Hilger, (**E**) *Cleome brachycarpa* Vahl ex DC., (**F**) *Brassica juncea* (L.) Czern., (**G**) *Gymnosporia royleana* Wall.ex M.A. Lawson, (**H**) *Cucumis maderaspatanus* L., (**I**) *Euphorbia prostrata* Aiton, (**J**) *Grewia asiatica* L., (**K**) *Bambusa vulgaris* Schrad. ex J.C.Wendl., (**L**) *Pongamia pinnata* (L.), (**M**) *Vachellia nilotica*, (L.) P.J.H.Hurter & Mabb., (**N**) *Echinochloa colonum* (L.) Link, (**O**) *Morus serrata* Roxb. (**P**) *Withania somnifera* (L.) Dunal, (**Q**) *Physalis angulata* L., (**R**) Solanum virginianum L., (**S**) *Zygophyllum creticum* (L.) Christenh. & Byng, (**T**) *Peganum harmala* L., and (**U**) *Malvastrum coromandelianum* (L.). (These are original photographs, Jabeen 2022).

The cross-regional analysis revealed that the current study also uncovered 21 unique WFVs that were rarely documented for human consumption in the studied ethnobotanical literature of Pakistan (Figure 6), i.e., *Ehretia obtusifolia*, *Euploca strigosa*, *Brassica juncea*, *Cleome brachycarpa*, *Gymnosporia royleana*, *Cucumis maderaspatanus*, *Croton bonplandianus*, *Euphorbia prostrata*, *Vachellia nilotica*, *Pongamia pinnata*, *Grewia asiatica*, *Malvastrum coromandilianum*, *Morus serrata*, *Argemone mexicana*, *Bambusa vulgaris*, *Echinochloa colonum*, *Solanum virginianum*, *Physalis angulata*, *Withania somnifera*, *Zygophyllum creticum*, and *Peganum harmala* (Figure 6). In addition, 14 WFVs were recorded in the current study for their novel uses and five WFVs for their novel edible parts used by the inhabitants of the area, reflecting the uniqueness of the flora and food culture of Pipli Pahar.

Our study highlights previously overlooked plant species, traditional practices, and cultural significance while revealing unique aspects of ethnobotanical knowledge in each area studied. In addition, we set ourselves apart from other studies by comparing our findings with those of previous studies in the region to highlight both the similarities and differences and to expand our understanding of ethnobotanical patterns and cultural practices. Despite the geographical and climatic differences between the regions mentioned, it is remarkable that they are similar in terms of certain food crops. Moreover, these regions belong to the same biogeographical zone, which suggests a certain ecological continuity and common botanical characteristics. Even if we recognize the geographical and climatic differences, the comparison is relevant due to the presence of similar species and the common biogeographical context.

## 3. Materials and Methods

### 3.1. Study Area

The research was conducted in the Pipli Pahar Plantation, which is a union of the Tehsil Depalpur in District Okara [62]. The study area is indicated in the map (Figure 1), which was self-created with ArcMap 10.7.1 software. Before the area was converted into an irrigated plantation, it was a typical dry tropical thorn forest described as a “Punjab Rakh.” Whereas, by notification No. 659, dated 8January-1931, the Governor in Council declared to constitute a reserved forest in a specified area in the Depalpur Tehsil of the Montgomery District. The Depalpur plantation was not managed regularly until the writing of the first exclusive working scheme for each unit during 1935–1936. On 30 April 1955, this land was transferred from the control of the Colonization Department to the Forest Department to part of the Depalpur Reserved Forest. Pipli Pahar is one of the eight boundaries of the Depalpur Reserved Forest, which is located at the end of the Depalpur Canal System (Sutlej Valley project). This is divided into 4 parts: Depalpur Plantation Cpt. No.96 Bed Nursery, Depalpur Nursery Cpt.95 Bed Nursery, Depalpur Plantation Cpt.133 (10 Ac), and Depalpur Plantation Cpt. No.94/A Bed Nursery. It is not a popular area because there is no modern development in the area [63].

**Figure 7:**
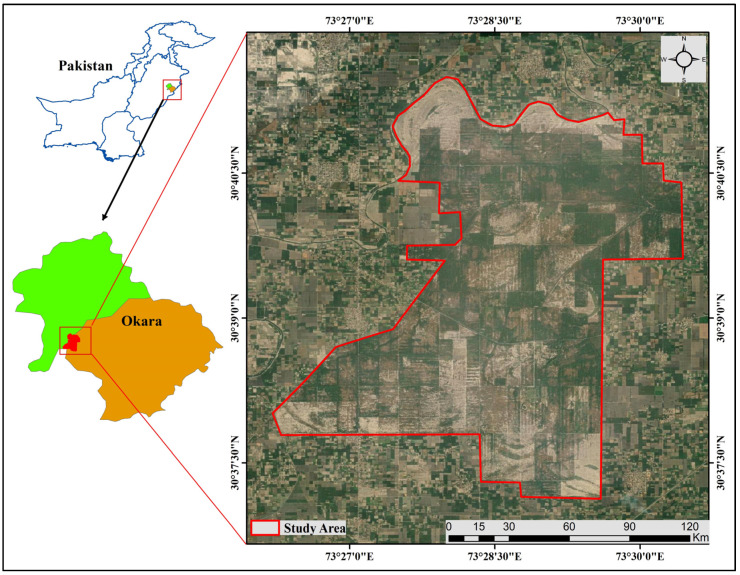
Map of the study area Pipli Pahar reserved forest.

The study area is almost 21.6 km away from Okara City and has a population of 110,427 [64]. The soil is shallow to deep and dark gray to brown in color. Summers are very hot and long, lasting from June to August when the temperature can exceed 40 °C. Winters are also harsh, with December and January being the coldest months when the temperature remains below 10 °C for many days (Figure 7).

### 3.2. Ethnobotanical Survey and Data Collection

From December 2020 to August 2021, various field visits, inspections, and meetings with the local population were carried out to collect the necessary data. Prior to conducting the field visits, the verbal consent of the informants and the formal written consent of the forest authorities were obtained. Information on the economic use of WFVs was collected using Participatory Rural Appraisal (PRA) based on discussions with local people and direct observations in selected fields [65]. The ethical guidelines of the International Society of Ethnobiology, 1988 [66] were followed throughout the survey. The survey design was approved by the Ethical Review Committee of the University of Okara (approval number UOERC#89). Informants were interviewed using a semi-structured and open-ended questionnaire [67], with questions asked in Urdu (the national language of Pakistan) and Punjabi (the mother tongue and regional language of Punjab). The purpose of the study was briefly explained to the respondents before collecting the required information from them. Forest guards and other forest department staff, including the gardeners of Shahbaz Sharif Forest Park (a park in the Pipli Pahar Reserved Forest), assisted in collecting the required plant samples. The residents of the study area were asked questions about various recipes for traditional food preparations and the use of WFVs in medicine. The forest officials also provided information on the cultivation and marketing of wild fruit trees and vegetable plants.

The collected specimens were identified using the available identification keys in the Flora of Pakistan [68,69]. For botanical names and related families, the method of the Angiosperm Phylogenic group [70] was used. The World Flora Online (2022) [71] and the Plant List (2010) [72] were also used for the nomenclature of the plants. The identified plant specimens were assigned with voucher numbers and deposited in the Botanical Herbarium at the University of Okara for future reference.

### 3.3. Data Analysis

As several qualitative parameters were recorded for the plants, it was necessary to evaluate this information using different indices (see below) to highlight their complementary or contrasting application. There are certain methodological differences for each index. Different formulas were used for each index, using different information from the qualitative surveys. All of these indices are important for converting qualitative observations into quantitative observations. We can then quantitatively determine which plants were more familiar to the residents (by RFC), which plant is used more (by UV), and which disease is predominantly treated with these plants (by ICF). The Jaccard index is a novelty index; it indicates an overall comparable uniqueness and authenticity of the data compared to the published articles from different aligned fields. The FIV and FUV indices provide useful information about the families; however, the fundamental difference between them is that the FIV indicates which family is more familiar to residents, while the FUV indicates which family has greater diversity in their utilization patterns. These data values were then organized in Microsoft Excel (2019) before being analyzed using SPSS (IBM 25) to obtain descriptive statistics. A Venn diagram was created in R 4.2.1 software to show overlap and variable characteristics.

#### 3.3.1. Relative Abundance

The relative abundance of the listed herb species was estimated using a visual estimation method [73]. For this purpose, an equal number and size of plots were randomly selected in each study area, and then the presence of each medicinal plant was counted and noted. The following formula was used to calculate the percentage relative abundance.

$$\frac{Total\;percentage\;cover\;of\;species\;in\;all\;plots}{Number\;of\;plots\;estimated}\times 100$$

All plants were categorized into different groups such as abundant, rare, frequent, common, and occasional by using the scale of relevant abundance, i.e., <5% (rare), 5–20% (occasional), 20–50% (frequent), 50–90% (common), and 90–100% (abundant).

#### 3.3.2. Family Use Values (FUV)

This index calculates the use value of a family by using the following formula [30].

$$FUV=\frac{\sum UVs}{NS}$$

(where, FUV = Family Use Value, ∑ UVs = Sum of the Use Values of all the species that are quoted from a family; NS = Total number of species that are quoted from the family).

#### 3.3.3. Family Importance Value (FIV)

The FIV is obtained by dividing the number of informants who mentioned the use of species by the total number of informants and then multiplying it by 100. It is an index that represents the importance of a family in a community, and it determines the consensus between the informants and the cited families [27].

$$FIV=\frac{\sum FCs}{N}\times 100$$

(where, FIV = Family Importance Value, FC = Total Number of Informants for a Family, and N = Total Number of Informants).

#### 3.3.4. Relative Frequency of Citation (RFC)

This index is obtained by dividing the number of informants who mentioned the use of species by the total number of informants who participated in the survey. The plant that was most popularly used received the highest RFC, and the plant species that were less used received a smaller number of citation frequencies in the community. This was calculated by using the following formula:

$$RFC=\frac{FCs}{N}$$

(where RFC = Relative Frequency of Citation, FCs = Number of informants who mentioned the use of fruit and vegetable species, and N = Total Number of informants) [30].

#### 3.3.5. Use Value (UV)

The UV counted the significance of each plant species based on the number of various uses that had been reported. The objective was to evaluate the significance of species in the community. It was calculated using the following formula:

$$UV=\frac{\sum Ui}{N}$$

where UV = Use Value for the species, ∑ Us = Sum of all the uses that were mentioned for a species, and N = Number of informants; here, N is not the total number of informants, it is the number of informants for a species in FC [30].

#### 3.3.6. Jaccard index (JI)

The cross-culture analysis to find the similarities or differences in indigenous knowledge among different cultural communities was determined by estimating the JI by the following formula:

$$JI=C\times \frac{100}{\left(a+b\right)}-c$$

where a is the species of the study area, b is the species recorded from the allied area, and c is the common species in both areas [74]. The % for the part used and the food dish recipe were also calculated to find the unique utilization of wild edible plants for their parts and traditional recipes.

## 4. Conclusions

This is the first documentation of traditional knowledge (TK) in the mentioned area, as no literature on WFVs has been published so far. According to Łuczaj [25], descriptive ethnobotanical studies are necessary for the documentation of disappearing traditional knowledge (TK) and represent a primary goal of ethnobiological and ethnobotanical research today. It is encouraging that 21 WFVs appear to be new as they have rarely been documented for human consumption in the studied ethnobotanical literature of Pakistan. Moreover, 14 out of 65 WFVs are described as novel uses, and 5 out of 65 WFVs are described as novel edible parts in the current study. The WFVs of the Pipli Pahar region have great economic value, but the trend of their utilization is declining because people are not aware of the utilization and value of these resources. In addition, low production and difficult cultivation conditions may be other factors for the “lost” uptake and propagation of these rare but valuable plants. These data can be used for the sustainable growth of WFV crops on a commercial scale. Their use can be promoted in our households and in the culinary industry to reduce the burden of conventional food crops. Furthermore, their nutraceutical properties can be evaluated for the discovery of alternative medicines. Local people can not only earn a living but also spread their culture through the use of WFVs in the culinary tourism industry. By classifying plant species into systematic units, we are not only documenting valuable traditional knowledge but also laying the foundation for future studies to build upon. This classification is crucial for recognizing patterns of plant use, distribution, and cultural significance in order to make informed decisions about conservation measures, sustainable resource management, and the preservation of traditional knowledge. The traditional knowledge of the population about the use of edible wild plants could be promoted through education and training. This could contribute to the conservation of natural resources and the optimal use of plants for domestic consumption. Such efforts can also improve the economy of local communities in this and other similar areas of the world.

## 5. Recommendations

To find better ways to conserve and effectively utilize WFVs, it would be helpful if the government could be persuaded to direct and fund the forest department and motivate local farmers to sustainably grow these WFV crops on a commercial scale. In addition, the owners of restaurants and pharmaceutical companies should also be encouraged to promote the wonderful potential of these WFVs to increase their interest in the production, consumption, and marketing of WFVs.

It is recommended to promote the use of traditional WFV-based dishes in culinary tourism to boost the economy and promote Pipli Pahar as a place for specialty tourism. For this purpose, food festivals or fairs should be organized in the Shahbaz Sharif Forest Park, which is located in the Pipli Pahar Reserved Forest. This park is already a reason for the popularity of the study area. To achieve this goal, dishes made from wild fruits and their nutritional benefits should be publicized through advertisements in print and electronic media. The locals should be encouraged to participate in festivals to preserve the traditional serving methods and flavors. This will also help to increase the household income of the local population. It is also recommended that coordinated research be conducted on edible wild fruit trees that can be used as a source of timber and honeybees so that the full potential of WFV trees can be realized.

The integration of alien species into the local diet as a source of wild fruits and vegetables (WFVs) is a complex process influenced by ecological, cultural, and socio-economic factors. Ecologically, the introduction of species such as *Argemone mexicana*, *Avena sativa*, *Bambusa vulgaris*, *Physalis minima*, *Lantana camara*, and *Zygophyllum creticum* occurs through different pathways favored by human activities and climate change. Culturally, acceptance depends on culinary traditions and taste preferences. Socio-economic factors such as market demand and land-use changes also play a role. Our study highlights the presence of these alien species among WFVs and emphasizes their importance for local diets. However, their integration raises concerns for native biodiversity and cultural heritage. Future research should assess the ecological impact and nutritional value of alien species and promote sustainable harvesting practices.

## Figures and Tables

**Table 1:** Demographic information of informants.

Gender	Number of People	%
Women	34	48.6
Men	36	51.4
**Ethnicity**		
Wattoo	5	7.1
Raheema	4	5.7
Qureshi	8	11.4
Mughal	7	10.0
Khokhar	7	10.0
Jhakhar	5	7.1
Nonari	5	7.1
Sheikh	6	8.6
Siddiqi	4	5.7
Baloch	4	5.7
Mahaar	6	8.6
Bhatti	4	5.7
Miyan	3	4.3
Kharal	2	2.9
**Age Groups**		
Between 20 and 40 years	25	35.7
Between 40 and 60 years	35	50.0
Above 60	10	14.3
**Level of Education**		
Illiterate	25	35.7
Elementary school	20	28.6
Matura	14	20.0
Middle school	5	7.1
University degree	4	5.7
Postgraduate or higher	2	2.9
**Occupation**		
Farmers	12	17.1
Shepherds	22	31.4
Gardeners	13	18.6
Forest guards	5	7.1
Forest officers	2	2.9
Teachers	2	2.9
Animal keeper	6	8.6
Sellers	8	11.4

**Table 2:** Comparative datasets for the number of taxa for dominant plant families, FIV (Family Importance Value) based on the ratio of the total number of informants who described them, and FUV (Family Use Value) based on the ratio of the total use for that plant family.

Family	Number of Taxa	FIV	FUV
Aizoaceae	1	39	1.11
Amaranthaceae	1	93	1.77
Arecaceae	1	100	5.00
Asteraceae	4	129	1.56
Boraginaceae	5	211	2.41
Brassicaceae	1	96	4.22
Cannabaceae	1	93	2.92
Capparaceae	2	106	2.43
Celastraceae	1	4	1.67
Chenopodiaceae	2	200	1.88
Cucurbitaceae	4	266	1.92
Euphorbiaceae	2	9	1.33
Fabaceae	6	499	2.41
Malvaceae	4	129	1.77
Meliaceae	1	100	4.50
Moraceae	7	573	2.24
Moringaceae	1	90	4.08
Myrtaceae	1	100	4.14
Oxalidaceae	1	83	2.60
Papaveraceae	1	11	1.25
Poaceae	3	234	2.06
Polygonaceae	2	110	1.05
Portulacaceae	1	71	1.60
Rhamnaceae	2	200	3.14
Rutaceae	1	69	1.63
Salvadoraceae	2	80	1.65
Solanaceae	4	357	1.69
Verbenaceae	1	57	1.00
Zygophyllaceae	2	34	1.14

## Data Availability

Voucher specimens were submitted to the Herbarium of the University of Okara Pakistan for forthcoming uses (Table 1).

## Appendix A

**Table A1:** A catalog of wild fruits and vegetables (WFVs) to highlight their different uses by the inhabitants of Pipli Pahar and possible references for their nutritional contents.

Family	Scientific Names	Herbarium Vouchers	Species Code	Local Name	Growth Form	Collection Time	Use Form	Edible Part	Mode of Utilization and Recipes of Local Cuisines	Medicinal Uses	FC	RFC	UV
**Aizoaceae**	*Trianthema portulacastrum* L.	UOBH00290	Tria.por	It Sit	Herbaceous	Dec–Nov	Vegetable	Whole Plant	(1) Shoots are cooked in saag (shoots of the plants mixed with *Brassica campestris* and Spinacia oleracea are boiled in water and then fried in oil with onion, garlic, ginger, green chilly with spices). (2) Sharbat (herbal tea) is prepared by boiling the plant in water with sugar.	Boils and acne, female infertility.	27	0.39	1.11
**Amaranthaceae**	*Digera muricata* (L.) Mart.	UOBH00291	Dige.mur	Tandla	Herbaceous	Aug–Feb	Vegetable	Shoots	(1) Cooked in saag (Shoots of the plants mixed with *Brassica campestris* and *Spinacia oleracea* are boiled in water and then fried in oil with onion, garlic, ginger, green chilly with spices). (2) It is also cooked with a mixture of ground chickpea lentils and potato by frying in onion, garlic, ginger, tomato, red chili, and spices.	Cooling effect on the body. Treats external cuts and wounds.	65	0.93	1.77
**Arecaceae**	*Phoenix sylvestris* (L.) Roxb.	UOBH00292	Phoe.syl	Khajoor	Tree	Jun–Aug	Fruit	Fruits	(1) Ripened fruits are eaten raw, especially during Iftar in Ramzan. (2) Halva is made (fruits of the plants are boiled in water or milk with other dry fruits, and then sugar is added to make a sweet dish). (3) Khajoor shake is prepared by mixing the fruits of *Phoenix sylvestris* with other domestic fruits and then adding sugar to make a (sweet energy drink).	Energetic, cooling effect on the body, treats Ulcers.	70	1	5
**Asteraceae**	*Carthamus oxyacantha* M.Bieb.	UOBH00293	Cart.oxy	Pohli	Herbaceous	Feb–Jul	Vegetable	Whole Plant	(1) Leaves with onion, garlic, ginger, and spices are cooked to make salan. (2) The whole plant is boiled in water to prepare sharbat (herbal tea).	Toothache and indigestion in children, warmness, swelling, cholera.	5	0.07	1.2
	*Cichorium intybus* L.	UOBH00294	Cich.int	Kasni	Herbaceous	Jun–Feb	Vegetable	Shoots	(1) Cooked in saag (Shoots of the plants mixed with *Brassica campestris* and *Spinacia oleracea* are boiled in water and then fried in oil with onion, garlic, ginger, green chili with spices).	The appetizer is effective against liver and stomach diseases.	70	1	2.36
	*Launaea procumbens* (Roxb.) Ramayya & Rajagopal	UOBH00295	Laun.pro	Bhattal	Herbaceous	Dec–Oct	Vegetable	Whole Plant	(1) Shoots are cooked in saag and salan. (2) Roots are cooked in salan.	Joints pain.	10	0.14	1.1
	*Sonchus oleraceus* (L.) L.	UOBH00296	Sonc.ole	Dhudhak booti	Herbaceous	Dec–Oct	Vegetable	Shoots	(1) Fresh shoots were eaten raw. (2) Cooked in saag (shoots of the plants mixed with *Brassica campestris* and *Spinacia oleracea* are boiled in water and then fried in oil with onion, garlic, ginger, green chilly with spices).	Leaf sap relieves earache. Febrifuge and tonic.	5	0.07	1.6
**Boraginaceae**	*Cordia dichotoma* G.Forst.	UOBH00297	Cord.dic	Lasoora	Tree	Mar–Jul	Fruit	Fruits	(1) Ripened fresh fruits are eaten raw. (2) Unripe fruits are used to make Achaar (Pickle). (3) Unripe fruits are ground with spices to prepare chutney (sauce).	Diarrhea, Hepatitis, sexual weakness in males.	70	1	4.03
	*Cordia myxa* L.	UOBH00298	Cord.myx	Lasoori	Tree	Mar–Jul	Fruit	Fruits	(1) Ripened fresh fruits are eaten raw. (2) Unripe fruits are used to make achaar (pickled).	Effective against cough and flu.	70	1	3.46
	*Ehretia acuminata* R.Br.	UOBH00299	Ehre.acu	Panna	Tree	Jan–Apr	Fruit	Fruits	(1) Ripened fresh fruits are eaten raw. (2) Unripe fruits are used to make Achaar (pickled).	Stomach and Liver disorders.	4	0.06	1.25
	*Ehretia obtusifolia* Hochst. ex A.DC.	UOBH00300	Ehre.obt	Gondhi	Shrub	Jan–Apr	Fruit	Fruits	(1) Ripened fresh fruits are eaten raw.	Sore throat.	3	0.04	1.33
	*Euploca strigosa* (Willd.) Diane & Hilger	UOBH00301	Heli.str	Bari Pudna	Herbaceous	Jun–Nov	Vegetable	Shoots	(1) chutney is prepared and served with food as sauce.			1	0.01
**Brassicaceae**	*Brassica juncea* (L.) Czern.	UOBH00302	Bras.jun	Jangli Sarsoon/Toriya	Herbaceous	Dec–Apr	Vegetable	Shoots/Seeds	(1) Cooked in saag (shoots of the plants mixed with *Brassica campestris* and *Spinacia oleracea* are boiled in water and then fried in oil with onion, garlic, ginger, green chilly with spices). (2) Seed oil is used to apply to hair. (3) Seed oil is also used in the preparation of pickles.	Hepatitis, Blood deficiency, Hair nourishment.	67	0.96	4.22
**Cannabaceae**	*Cannabis sativa* L.	UOBH00303	Cann.sat	Bhung	Herbaceous	Jul–Oct	Vegetable	Leaves	(1) Ghota (herbal drink) is prepared by grinding the leaves.	Cooling effect on body, Laxative.	65	0.93	2.92
**Capparaceae**	*Capparis decidua* (Forssk.) Edgew.	UOBH00304	Capp.dec	Dailay/Kariya	Shrub	Aug–Nov	Fruit	Fruits	(1) Unripe fruits are used to make achaar (pickled). (2) Ripened fresh fruits are eaten raw. (3) Ripened fruits are used to make murabba (jam).	Blood pressure, Liver disorders.	70	1	3.86
	*Cleome brachycarpa* Vahl ex DC.	UOBH00305	Cleo.bra	Bophali/Dophali	Herbaceous	Jul–Mar	Vegetable	Whole Plant	(1) A cold drink is prepared by soaking the plant in water and taken as sharbat.	Stomach disorders.	4	0.06	1
**Celastraceae**	*Gymnosporia royleana* Wall.ex M.A. Lawson	UOBH00306	Gymn.roy	Pataki/Karonda	Tree	Feb–May	Fruit	Fruits	(1) Unripe fruits are used to make achaar (pickled). (2) Ripened fruits are used to make murabba (jam). (3) Ripened fresh fruits are eaten raw.	Joints pains.	3	0.04	1.67
**Chenopodiaceae**	*Chenopodium album* L.	UOBH00307	Chen.alb	Bathoo	Herbaceous	Jan–Dec	Vegetable	Shoots	(1) Cooked in saag (shoots of the plants mixed with *Brassica campestris* and *Spinacia oleracea* are boiled in water and then fried in oil with onion, garlic, ginger, green chilly with spices).	Cooling effect on the body, treats boils.	70	1	2.59
	*Chenopodiastrum murale* (L.) S.Fuentes, Uotila & Borsch	UOBH00308	Chen.mur	Bathu/Karonda/Chulai	Herbaceous	Jan–Dec	Vegetable	Shoots	(1) Cooked in saag (shoots of the plants mixed with *Brassica campestris* and *Spinacia oleracea* are boiled in water and then fried in oil with onion, garlic, ginger, green chilly with spices).	Joints pains.	70	1	1.17
**Cucurbitaceae**	*Citrullus colocynthis* (L.) Schrad.	UOBH00309	Citr.col	Tumma/Kor Tumma	Herbaceous	Aug–Nov	Vegetable	Fruits/Seeds	(1) Unripe fruits are used to make Achaar (Pickle). (2) Ripened fruits are used to make murabba (jam). (3) Seeds are used to make phakki (powder). (4) Fruit powder is mixed with the powder of Shirin’ seed powder, Kalwanji, and gum of Keekar in butter to make *goli* (herbal tablet). (5) Spice for animals. (6) Fruits are crushed and under the feet to treat the joint pain.	Phakki for abdominal pains and worms. Goli for diabetes. Joints pain.	60	0.86	3.17
	*Cucumis melo var. agrestis* Naudin	UOBH00310	Cucu.mel	Chibherr	Herbaceous	Jul–Oct	Fruit/Vegetable	Fruits	(1) Ripened fresh fruit is eaten raw, and (2) chatni (sauce) is prepared by crushing the fruits and seeds with onions. (3) Ripened fruits are also cooked in salan.	Moisturizes the skin, Useful for digestion and acidity.	70	1	1.97
	*Momordica balsamina* L.	UOBH00311	Momo.bal	Jangli Karela	Herbaceous	May–Oct	Vegetable	Fruits	(1) Fruits are sometimes used to make salan by mixing it with chickpea lentils or qeema of chicken or beef.	Diabetes	50	0.71	1.2
	*Cucumis maderaspatanus* L.	UOBH00312	Muki.mad	Chaina Chibherr	Herbaceous	Jul–Oct	Fruit	Fruits	(1) Ripened fresh fruits are eaten raw.	Digestion	6	0.09	1.33
**Euphorbiaceae**	*Croton bonplandianus* Baill.	UOBH00313	Crot.bon	Langrol	Herbaceous	Jul–Nov	Vegetable	Whole Plant	(1) Plant is boiled in water and then sugar is added to make sharbat (herbal tea).	Wounds, Joints infections.	3	0.04	1
	Euphorbia prostrata Aiton	UOBH00314	Euph.pro	Hazardani	Herbaceous	Dec–Nov	Vegetable	Shoots	(1) Fresh shoots are eaten raw.	Lose motions.	3	0.04	1.67
**Fabaceae**	*Vachellia nilotica* (L.) P.J.H.Hurter & Mabb.	UOBH00315	Acac.nil	Desi Keekar	Tree	Jun–Dec	Fruit	Pods/Flowers/Gum	(1) Unripe pods are used to make achaar (pickled). (2) Gum (locally called *cheer*) eaten raw as chewing gum. (3) Gum is mixed in water to make sharbat (drink) by mixing it in water. (4) Flowers are boiled in water to make sharbat (herbal tea). (5) Flowers are cooked insalan.	Leucorrhea, Cooling effect on body.	70	1	2.67
	*Bauhinia variegata* L.	UOBH00316	Bauh.var	Kachnar	Tree	Feb–Jul	Vegetable	Leaves/Buds/Pods	(1) Buds and leaves are sometimes cooked in salan by mixing them with meat. (2) Unripe buds and pods are used to make achaar (pickled).	Common fever and pneumonia.	68	0.97	4.29
	*Cassia fistula* L.	UOBH00317	Cass.fis	Amaltas/Ambartash	Tree	Feb–Jul	Fruit/Vegetable	Fruits/Leaves/Flowers/Seeds	(1) Ripened fresh fruits are eaten raw. (2) Fruits are also cooked meat. (3) Leaves and flowers are cooked in saag. (4) Flowers are used to prepare *gulkand*.	Alimentary tract infections in children.	65	0.93	3.62
	*Pongamia pinnata* (L.) Pierre	UOBH00318	Pong.pin	Karunjwa	Tree	Feb–Jan	Vegetable	Leaves	(1) Leaves are used to make chutney (sauce).	Fever	8	0.11	1
	*Prosopis cineraria* (L.) Druce	UOBH00319	Pros.cin		Tree	Jun–Sep	Fruit	Pods	(1) Unripe pods are used to make Achaar (Pickle).		69	0.99	1.06
	*Neltuma juliflora* (Sw.) Raf.	UOBH00320	Pros.jul	Pahari Keekar/Jund/Arjun	Tree	Jun–Sep	Fruit	Pods	(1) Unripe pods are used to make achaar (pickled). (2) Pods are also cooked in salan with other vegetables such as potatoes.	Antibiotic, anti-fertility, asthma.	69	0.99	1.81
**Malvaceae**	*Grewia asiatica* L.	UOBH00321	Grew.asi	Falsa	Shrub	Apr–Aug	Fruit	Fruits	(1) Ripened fresh fruits are eaten raw. (2) Fruits are also ground to make sharbat (sweet drink).	Cooling effect on the body.	68	0.97	4
	*Grewia tenax* (Forssk.) Fiori	UOBH00322	Grew.ten	Phalsa/Ganger	Shrub	Mar–Jun	Fruit	Fruits	(1) Ripened fresh fruits are eaten raw.	Diarrhea.	3	0.04	1
	*Malvastrum coromandelianum* (L.) Garcke	UOBH00323	Malv.cor	Cheri Choga	Herbaceous	Nov–Dec	Vegetable	Seeds	(1) Unripe seeds are eaten raw.		4	0.06	1
	Malva parvifloraL.	UOBH00324	Malv.par	Sonchal/Khubazi	Herbaceous	Jul–Oct	Fruit/Vegetable	Fruits/Leaves	(1) Ripened fruits are eaten raw. (2) Leaves are cooked in salan or saag.	Urinary problems.	15	0.21	1.07
**Meliaceae**	*Azadirachta indica* A.Juss.	UOBH00325	Azad.ind	Naim/Neem	Tree	Feb–Jul	Fruit	Fruits/Leaves	(1) Ripened fresh and dried fruits are eaten raw. (2) Leaves are crushed and mixed in water to make sharbat (herbal drink).	Anti-bacterial for wounds, blood purifier.	70	1	4.5
**Moraceae**	*Ficus benghalensis* L.	UOBH00326	Ficu.ben	Boher	Tree	Mar–Jul	Fruit	Fruits	(1) Ripened fresh fruits are eaten raw.	Cooling effect on the body. Body weakness, male sexual weakness.	66	0.94	1.44
	*Ficus palmata* Forssk.	UOBH00327	Ficu.pal	Jangli Injeer	Tree	Mar–Jul	Fruit	Fruits	(1) Ripened fresh fruits are eaten raw.	Tonsils, Pneumonia, kidney infections	55	0.79	1.05
	*Ficus racemosa* L.	UOBH00328	Ficu.rac	Gulhar	Tree	Mar–Jul	Fruit	Fruits	(1) Ripened fresh fruits are eaten raw.	Sexual weakness, constipation.	7	0.1	1.43
	*Ficus religiosa* L.	UOBH00329	Ficu.rel.	Peepal	Tree	Mar–Jul	Fruit	Fruits	(1) Riepened fresh fruits are eaten raw.	Digestive.	63	0.9	1.03
	*Morus alba* L.	UOBH00330	Moru.alb	Toot	Tree	Feb–Apr	Fruit	Fruits	(1) Ripened fresh fruits are eaten raw.	Fruits are effective against throat infections.	70	1	2.86
	*Morus nigra* L.	UOBH00331	Moru.nig	Toot	Tree	Feb–Apr	Fruit	Fruits	(1) Ripened fresh fruits are eaten raw.	Throat infections, pre-mature graying of hairs.	70	1	3.94
	*Morus serrata* Roxb.	UOBH00332	Moru.ser	Shahtoot	Tree	Feb–Apr	Fruit	Fruits	(1) Ripened fresh fruits are eaten raw.	Blood deficiency, throat infections.	70	1	3.96
**Moringaceae**	*Moringa oleifera* Lam.	UOBH00333	Mori.ole	Suhanjna	Tree	Apr–Aug	Vegetable	Fruits/Leaves/Flowers/Seeds/Roots	(1) Ripened fresh fruits are cooked as in salan. (2) Unripe fruits and roots are pickled to make achaar. (3) Flowers and leaves are boiled in water to prepare *kehwa* (herbal tea).	Body weakness, kidney infections, diabetes, asthma.	63	0.9	4.08
**Myrtaceae**	*Syzygium cumini* (L.) Skeels	UOBH00334	Syzy.cum	Jaman	Tree	May–Jul	Fruit	Fruits	(1) Ripened fresh fruits are eaten raw. (2) Sharbat (sweet drink) is prepared by mixing paste of fruit pulp in water and sugar.	Cooling effect on body, Hepatitis, Blood deficiency.	70	1	4.14
**Oxalidaceae**	*Oxalis corniculata* L.	UOBH00335	Oxal.cor	Khatti Mithi/Khatti Tee	Herbaceous	Jan–Dec	Vegetable	Shoots	(1) Cooked in saag (Shoots of the plants mixed with *Brassica campestris* and *Spinacia oleracea* are boiled in water and then fried in oil with onion, garlic, ginger, green chilly with spices). (2) Shoots are crushed with coriander, green chili, and spices to make chutney (sauce). (3) Eaten raw.	Cooling effect on body, Hepatitis, heart diseases.	58	0.83	2.6
**Papaveraceae**	*Argemone mexicana* L.	UOBH00336	Arge.mex	Satyanasi/Dhatoora	Herbaceous	Apr–Jul	Vegetable	Whole Plant	(1) Sharbat (herbal tea) is prepared by boiling the plant in water.	Achene.	8	0.11	1.25
**Poaceae**	*Avena sativa* L.	UOBH00337	Aven.sat	Joun/Javi	Herbaceous	Jan–May	Vegetable	Seeds	(1) Flour of seeds is used to prepare *roti* (pudding). (2) Crushed seeds are boiled in water or milk with sugar to prepare *daliya* (sweet dish). (3) Crushed seeds are mixed in water with other ingredients to prepare *sattu* (sweet drink).	Lowers blood cholesterol level, cooling effect on the body.	64	0.91	3.78
	*Bambusa vulgaris* Schrad. ex J.C.Wendl.	UOBH00338	Bamb.vul	Waans/Baans	Grass	Jan–Dec	Vegetable	Roots	(1) Young soft branches are eaten raw. (2) Roots are used to make achaar (pickled).	Urinary bladder stones.	70	1	1.29
	*Echinochloa colonum* (L.) Link	UOBH00339	Echi.col	Swanakh Grass	Herbaceous	Mar–Oct	Vegetable	Seeds	(1) Seeds are cooked or ground into flour to make *roti* (bread).	Urinary bladder stones.	30	0.43	1.1
**Polygonaceae**	*Polygonum plebeium* R.Br.	UOBH00340	Poly.ple	Hind Rani/Laal Jhaari.	Herbaceous	Feb–Jul	Vegetable	Shoots	(1) Leaves and stems are cooked in salan with other vegetables. (2) Shoots are soaked in water to prepare sharbat (cold drink).	Cough, warmness, achene.	30	0.43	1.07
	*Rumex dentatus* L.	UOBH00341	Rume.den	Jangli Palak	Herbaceous	Nov–Jun	Vegetable	Shoots	(1) Cooked in saag (shoots of the plants mixed with *Brassica campestris* and *Spinacia oleracea* are boiled in water and then fried in oil with onion, garlic, ginger, green chilly with spices).	Blood deficiency, joint diseases.	47	0.67	1.04
**Portulacaceae**	*Portulaca oleracea* L.	UOBH00342	Port.ole	Bathuwan Booti	Herbaceous	Mar–Dec	Vegetable	Shoots	(1) Cooked in saag (shoots of the plants mixed with *Brassica campestris* and *Spinacia oleracea* are boiled in water and then fried in oil with onion, garlic, ginger, green chilly with spices).	Antiseptic.	50	0.71	1.6
**Rhamnaceae**	*Ziziphus mauritiana* Lam.	UOBH00343	Zizi.mau	Beri	Tree	Feb–Jun	Fruit	Fruits	(1) Ripened fruits are eaten raw. (2) Ripened fruits are dried for later use to make *joshanda* (herbal tea) with other dried herbs.	Cough, flu, sexual weakness in men.	70	1	3
	*Ziziphus nummularia* (Burm.f.) Wight & Arn.	UOBH00344	Zizi.nam	Saho Berri	Tree	Jan–Jun	Fruit	Fruits	(1) Ripened fruits are eaten raw.	Laxative, carminative, respiratory problems.	70	1	3.29
**Rutaceae**	*Bergera koenigii* L.	UOBH00345	Murr.koe	Kari Patta	Tree	Mar–Oct	Vegetable	Leaves	(1) Leaves are used as condiments.	Digestive, prevents graying of hairs, cholesterol.	48	0.69	1.63
**Salvadoraceae**	*Salvadora oleoides* Decne	UOBH00346	Salv.ole	Waan	Tree	Feb–Jun	Fruit	Fruits	(1) Ripened fresh fruits are eaten raw.	Gum and tooth diseases.	52	0.74	1.56
	*Salvadora persica* L.	UOBH00347	Salv.per	Pilu	Tree	Feb–Jun	Fruit	Fruits	(1) Ripened fresh fruits are eaten raw.	Appetizer, bowel functions, regulates the menstrual cycle.	4	0.06	1.75
**Solanaceae**	*Solanum nigrum* L.	UOBH00348	Sola.nig	Peelakan	Herbaceous	Dec–Aug	Fruit/Vegetable	Whole Plant	(1) Ripened fruits are eaten raw. (2) Leaves are cooked in *salan*. (3) Leaves are cooked in *saag* (shoots of the plants mixed with *Brassica campestris* and *Spinacia oleracea* are boiled in water and then fried in oil with onion, garlic, ginger, green chili with spices). (4) *sharbat* (herbal tea) is made by boiling the whole plant in water.	Hepatitis B and C, cooling effect on the body.	70	1	3.29
	*Solanum virginianum* L.	UOBH00349	Sola.xan	Kandyari	Herbaceous	Dec–Nov	Vegetable	Fruits/Leaves/Seeds	(1) Seeds are cooked in *karrhi* and served with *pakoras*. (2) Salan is made from fruits and leaves mixed with other vegetables. (3) Fruits are cut into four pieces, and then gram flour is filled in it to make pakoray.	Joints, infections, piles, back pain.	66	0.94	1.06
	*Physalis angulata* L.	UOBH00350	Phys.min	Bhamolay	Shrub	Dec–Apr	Fruit	Fruits	(1) Ripened fresh fruits are eaten raw.	Cooling effect on the body. Ant cancerous	64	0.91	1.2
	*Withania somnifera* (L.) Dunal	UOBH00351	With.som	Asgandh Nagori/Paner dodhi/Rankan	Shrub	Dec–May	Fruit	Fruits/Roots	(1) Ripened fresh fruits are added to milk to make yogurt. (2) Powder of roots is taken to increase height.	Digestive disorders, Immunity booster, joint pain, medicine for animals’ worms.	50	0.71	1.2
**Verbenaceae**	*Lantana camara* L.	UOBH00352	Lant.cam	Bhangi Booti/Panj Phali	Shrub	Apr–Jul	Fruit/Vegetable	Fruits/Leaves	(1) Ripened fresh fruits are eaten raw. (2) Leaves are boiled in water to make kehwa (herbal tea).	Cough, flu, carminative.	20	0.29	1
**Zygophyllaceae**	*Zygophyllum* creticum (L.) Christenh. & Byng	UOBH00353	Fago.cre	Dhamman	Herbaceous	Mar–Nov	Vegetable	Whole Plant	(1) Plant is dried and then ground to make powder, which is taken as *phakki*. (2) A cold drink is made by soaking the plant in water for the whole night and is drunk as sharbat. (3) Herbal tea is made by boiling the plant in water and then adding sugar and cheese. It is drunk as sharbat.	Cancer, Hepatitis, Female Infertility.	14	0.2	1.07
	*Peganum harmala* L.	UOBH00354	Peg.har	Harmal	Herbaceous	Feb–Oct	Vegetable	Whole Plant	(1) “*Kheer*” (sweet dish) is prepared by boiling the plant in water and then adding sheep’s milk with sugar.	Joints pain.	10	0.14	1.2
